# Reliability and Clinical Feasibility of Three Assessment Methods for Head and Neck Lymphedema in Head and Neck Cancer Patients

**DOI:** 10.3390/cancers17101672

**Published:** 2025-05-15

**Authors:** Kaat Van Aperen, Sandra Nuyts, Thierry Troosters, Nele Devoogdt, Tessa De Vrieze, Ceren Gürsen, Kaat Verbeelen, Johannes Devos, An De Groef

**Affiliations:** 1 Laboratory of Experimental Radiotherapy, Department of Oncology, University of Leuven, 3000 Leuven, Belgium; kaat.vanaperen@kuleuven.be (K.V.A.); sandra.nuyts@kuleuven.be (S.N.); 2 Department of Rehabilitation Sciences and Physiotherapy, University of Leuven, 3000 Leuven, Belgium; thierry.troosters@kuleuven.be (T.T.); nele.devoogdt@kuleuven.be (N.D.); tessa.devrieze@kuleuven.be (T.D.V.); ceren.guersen@kuleuven.be (C.G.); kaat.verbeelen@kuleuven.be (K.V.); 3 Department of Radiation Oncology, Leuven Cancer Institute, University Hospitals Leuven, 3000 Leuven, Belgium; 4 Respiratory Rehabilitation and Respiratory Division, University Hospitals Leuven, 3000 Leuven, Belgium; 5 Department of Physical Medicine and Rehabilitation, University Hospitals Leuven, 3000 Leuven, Belgium; 6 Centre for Lymphedema, Department of Vascular Surgery, University Hospitals Leuven, 3000 Leuven, Belgium; 7 MOVANT Research Group, Department of Rehabilitation Sciences and Physiotherapy, University of Antwerp, 2610 Antwerp, Belgium; 8 Department of Radiology, University Hospitals Leuven, 3000 Leuven, Belgium; johannes.devos@uzleuven.be

**Keywords:** head and neck cancer, lymphedema, assessment, reliability, feasibility

## Abstract

**Background/Objectives**: Head and neck lymphedema (HNL) is a common complication after head and neck cancer (HNC) treatment. Reliable and feasible assessment methods are essential for monitoring and management. This study aimed to evaluate the reliability and clinical feasibility of three methods for assessing external HNL in HNC patients: local tissue water (%) using the MoistureMeterD Compact (MMDC), neck circumference using a tape measure, and dermal thickness using B-mode ultrasound. **Methods**: Thirty-three HNC patients with potential HNL were included. Measurements were performed on the same day, twice by the same rater and once by a different rater. Intraclass correlation coefficients (ICC^2,1^), (relative) standard error of measurement ((%)SEM), smallest real difference (SRD), systematic differences across measurement occasions, and Bland–Altman plots with 95% limits of agreement were analyzed. Time efficiency and clinical limitations were assessed. As an exploratory analysis, Spearman correlations among methods were examined. **Results**: All methods demonstrated moderate to very strong reliability (ICCs^2,1^ 0.781–0.994), except dermal thickness (ICCs^2,1^ 0.136–0.354). Differences between raters and within one rater were not clinically meaningful. Neck circumference showed the highest reliability (ICCs^2,1^ 0.958–0.994) and was the fastest to perform with the fewest limitations. The methods showed weak correlations with each other. **Conclusions**: Neck circumference was the most reliable and time-efficient method assessing HNL in clinical practice but is limited to the neck region. For the head, local tissue water assessment seems the most reliable and feasible. The methods assess different aspects of HNL. Further research should confirm how these methods can complement each another.

## Simple Summary

This study investigates methods for assessing external head and neck lymphedema, a common and often debilitating condition that can occur after treatment for head and neck cancer. This study aimed to evaluate the reliability and clinical feasibility of three methods: local tissue water assessment, neck circumference assessment, and dermal thickness assessment. By testing these methods on head and neck cancer patients, the study aimed to determine which method is most reliable, easy to use, and suitable for clinical practice. The methods evaluated different aspects of HNL, suggesting that combining them may improve overall assessment of lymphedema in head and neck cancer patients. These findings ultimately lead to better patient care and more effective monitoring of this condition in clinical settings.

## 1. Introduction

Head and neck lymphedema (HNL) is a prevalent and debilitating side effect that often arises following treatment for head and neck cancer (HNC) [1,2,3,4,5]. Treatments such as surgical lymph node removal and radiotherapy can damage lymphatic pathways and surrounding tissues, disrupting lymph flow and resulting in lymphatic fluid accumulation, known as lymphedema [6,7,8,9]. Lymphedema can present externally, affecting the soft tissues of the face and neck, or internally within areas such as the oral cavity, pharynx, or larynx, or as a combination of both [10,11,12]. Reported prevalence rates for lymphedema show considerable variation, ranging from 12% to 90% for external HNL and reaching up to 97% for internal HNL [1,5,9,13,14]. This variability is likely influenced by differences in the timing of assessments across studies, with a trend of higher prevalence rates early after treatment. Patients with HNL may experience substantial symptom burden and face functional impairments [8,10,12,15,16,17].

Despite its large prevalence and impact, there is currently no consensus on a “gold standard” method for diagnosis, classification, or monitoring of HNL, which may also contribute to the wide variety in prevalence rates as well [14,18,19]. Starmer et al. summarized 38 different assessment methods in the literature used to evaluate HNL, which ranged from clinician rating scales to quality-of-life measures [19]. However, only 11 of these methods underwent validation and reliability testing, revealing varying levels of bias among them [19]. As highlighted in the review by Starmer et al. [19], the complexity of HNL and its significant impact suggest that a multifactorial assessment approach is most suitable. However, the specific components of such an assessment have yet to be defined. For this, based on the available literature, three assessment methods seem promising.

First, research in breast cancer-related lymphedema provides evidence of good clinometric properties for assessment methods for local tissue water [20]. To our knowledge, only a few studies investigated this method in the head and neck region [14,19,21]. The Assessment of Lymphedema of the Head and Neck (ALOHA) approach by Purcell et al. demonstrated strong inter-rater and intra-rater reliability, as well as strong known-groups validity for assessing local tissue water using a single measurement point in the submental region [22]. Furthermore, a significant correlation was reported between water content and a clinical rating scale based on visual inspection and palpation, supporting the validity of water content measurements [22]. Arends et al. investigated multiple measurement sites in the head and neck region for a more comprehensive evaluation [21]. Their assessment protocol showed strong to very strong test-retest reliability in a non-lymphedema population [21]. However, they did not investigate this protocol with multiple sites in a HNC population [21].

Second, tape measurements including neck circumference measurements, are currently the most commonly used assessment method for HNL in clinical practice [14,19]. Neck circumference measurements provide a fast and practical way to quantify swelling in this region, offering a standardized approach similar to that used for limb lymphedema [23]. Only two studies have reported reliability data on neck circumference measurements in the HNC population [14,19]. The ALOHA approach, with a lower and upper neck measurement, has demonstrated strong to very strong reliability in healthy controls and HNC patients with HNL [14,18,19]. The study by Chotipanich et al. showed good to excellent reliabilities for three circumferences of the neck (upper, middle, and lower). Given the frequent use of circumference measurements in daily practice, it is essential to further evaluate their clinical feasibility and reliability.

Third, determining dermal thickness by ultrasound is a reliable method to evaluate breast lymphedema [24]. Several studies involving breast cancer patients have already demonstrated strong construct validity, establishing dermal thickness as a significant clinical feature of breast lymphedema [24,25,26,27]. In the HNC population, ultrasound is an innovative and emerging method and has been infrequently reported as an assessment method for HNL [14,19]. In the systematic review by Fadhil et al., it was mentioned in only five studies, while the review by Starmer et al. included only three studies [14,19]. To the best of our knowledge, the reliability of this assessment method has not yet been investigated in the head and neck region. Further, as described above, external HNL is a complex phenomenon, illustrated by the high number of assessment methods found in the literature [14,19]. The extent to which the different constructs measured by the different methods relate to each other is not yet investigated [18]. Therefore, gaining insight into the intercorrelation of different assessment methods may improve understanding of the value of different assessment methods for diagnosis and monitoring of HNL.

In conclusion, all three assessment methods have demonstrated potential for assessing HNL. However, the reliability of the assessment of local tissue water at multiple measurement sites in HNC patients has never been investigated [18,21,22]. While the previous literature has highlighted the good reliability of neck circumference measurements as a traditional technique, inconsistencies in precision and reliability suggest the need for a deeper understanding of their clinical feasibility [14,19]. Furthermore, the reliability of dermal thickness assessment in the head and neck region remains unexplored [14,19]. In addition, reliability and the clinical feasibility of multiple assessment methods for HNL have never been examined together in one study [14,18,19]. Therefore, the aim of the present study is to investigate the inter- and intra-rater reliability, and clinical feasibility of these three external HNL assessment methods in HNC patients with potential HNL. In addition, how these different assessment methods of HNL relate to each other will be explored with correlation analyses.

## 2. Materials and Methods

### 2.1. Study Design

This cross-sectional sub-study is part of a large clinical trial, i.e., the EffEx-HN trial, which received ethical approval from the Ethics Committee of the University Hospitals Leuven (S65549). The sub-study was approved as an amendment to the protocol of the main trial. The main trial is registered at Clinicaltrials.gov (NCT05256238), and a detailed protocol has been published elsewhere [28]. This study was conducted in accordance with the Declaration of Helsinki and is reported following the recommended STROBE (STrengthening the Reporting of OBservational studies in Epidemiology) guidelines for observational studies [29].

### 2.2. Participants

Participants were recruited from the main EffEx-HN trial at the Oncology Department of UZ Leuven between April 2023 and February 2024 [28]. Participants of the EffEx-HN trial were diagnosed with a primary malignant tumor in the head and neck region (nasal cavity/paranasal sinuses, oropharynx, oral cavity, hypopharynx, larynx, salivary gland, nasopharynx, thyroid or other), and planned for curative (chemo)radiotherapy. This sub-study was offered as an additional assessment during one of the participants’ regular assessment time points, scheduled at 6 weeks, 12 weeks, 6 months, or 12 months after initiation of radiotherapy. This sub-study focused on a subset of participants from the main trial, selected based on their availability and willingness to participate in these additional assessments. Only patients who wished to keep their beard were excluded from the present study. All participants provided separate written informed consent specifically for this sub-study.

### 2.3. Procedures

All assessments were conducted by two doctoral researchers with a Master of Science in Physiotherapy and Rehabilitation sciences with two years of experience in the field of cancer-related lymphedema (KVA and KV). As part of the EffEx-HN trial, a single assessment was already scheduled and conducted by the first rater (R1, KVA). For the purposes of this sub-study on measurement reliability, two additional assessments were performed: one by a second rater (R2, KV) to evaluate inter-rater reliability, and a repeated assessment by the first rater (R1b) to evaluate intra-rater reliability. All assessments were conducted consecutively on the same day. The sequence of the three assessment methods was consistent across all participants, following the previously specified order (R1a, R2, R1b). The assessments were always performed from the right to the left side for practical reasons, i.e., the positioning of the ultrasound device in the room. Prior to the start of the study, the two researchers (KVA and KV) were trained in the assessment methods by colleagues in our research group familiar with the assessment methods (10 h). In addition, a head and neck radiologist (JD) was consulted to practice and interpret the ultrasound images.

### 2.4. Assessments

The assessment methods of interest included: (1) local tissue water (%), measured with the MoistureMeterD Compact (MMDC) device; (2) neck circumference (cm) with a tape measure; and (3) dermal thickness (µm) measured with B-mode ultrasound. Table 1 provides an overview of the protocol, including the measurement points in the head and neck region for each assessment method [21]. The standardized protocol for local tissue water and dermal thickness measurement is based on the research of Arends et al. and consists of 15 measurement points, of which 7 are bilateral and 1 submental [21]. Measurement points were marked with a washable skin pencil. Neck circumference was assessed only at the three measurement points in the neck area. Anatomical reference names were assigned to each measurement point, based on their positions relative to specific anatomical landmarks. The measurement points were erased between the three measurement sessions, but not between each assessment method. Participants refrained from smoking or consuming hot beverages for at least 60 min prior to the measurements. Participants were asked to arrive without makeup or rich moisturizers on their skin, and men were requested to have smoothly shaved skin. The participants were positioned upright on the bed, lying supine with their head maintained at approximately a 90° angle relative to the headrest. The head was positioned neutrally, without a pillow. Arms and legs were stretched out neutrally, although a rolling pillow was used under the knees for comfort.

**Figure 1:**
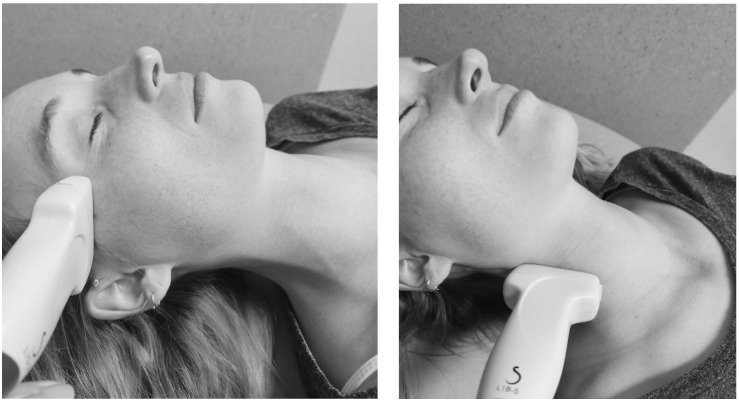
Positioning of the L10-2 probe on the head, and the L18-5 probe in the neck.

First, the % local tissue water was measured with the MMDC (Delfin Technologies Ltd., Kuopio, Finland). The MMDC device measures the tissue dielectric constant (TDC), which is then converted into a percentage of water content. The MMDC device has an effective measurement depth of 2.5 mm, measuring the superficial dermis and (upper) subcutis. The MMDC was positioned perpendicular to the skin with minimal pressure, using the “spot” mode. A single measure was expressed in %, without decimals. An average of three measures was made and used for analysis. Second, for the circumference measurement of the neck, the three predetermined measurement points in the neck area (5a, 5b, and 5c) and a tape measure were used. The tape measure was positioned perpendicular to the longitudinal axis of the neck. The measurements were taken once at each measurement point. The outcomes were expressed in cm, rounded to the nearest 0.50. Third, the dermal thickness was measured with B-mode ultrasound. The dermis is located between the epidermis and subcutis [30]. On B-mode ultrasound the dermis is the intermediate gray band between the hyperechoic epidermis and the hypoechoic subcutaneous tissue [30]. The ‘SuperSonic MACH30’ (Hologic Inc., Marlborough, MA, USA, software version 3.0) was used. An L10-2 probe, with a 2–10 MHz bandwidth, was used for the measurement points on the face (1, 2, 3a, 3b, and 4). An L18-5 probe, with a 5–18 MHz bandwidth, was used for the measurement points 5a-c in the neck region. The probes were consistently positioned perpendicular to the skin and the center of the probe was placed at the measurement point. In facial measurements, the probe was placed horizontally, while in neck measurements, it was positioned longitudinally along the sternocleidomastoid muscle. The positioning of the probes is shown in Figure 1 and clarified with a red rectangle in Table 1. Three images at each measurement point (at 15 locations) were taken and the mean thickness (μm) was used for analyses. The images were exported as DICOM files and uploaded into the ‘Weasis’ program (v4.2.1) for dermal thickness measurement, which was performed by the first rater (KVA).

In addition, patient characteristics were extracted from patients’ electronic medical files and entered into the Research Electronic Data Capture system (REDCap) [31]. The collected data included sociodemographic information (age, time since initiation of radiotherapy, gender, and skin type) and medical information (primary tumor location, TNM classification, and cancer treatment). The TNM classification was determined according to the guidelines outlined in the Quick Reference Guide to TNM Staging of Head and Neck Cancer and Neck Dissection Classification [32]. Body height was measured with a stadiometer, and body weight was measured with the InBody 770 device, as part of the original clinical trial [28]. In addition, body mass index (BMI) was calculated with the InBody 770 device. The presence of subjective HNL was determined by two subjective methods: (1) item 6 of the Lymphedema Symptom Intensity and Distress Survey-Head and Neck version 2.0 (“Presence of swelling in your face, head or neck”) and (2) visual inspection performed by the first rater (R1, KVA). These methods were part of the standard procedures of the original clinical trial [28].

### 2.5. Statistical Analyses

In accordance with the recommendations of Shrout and Fleiss for reliability studies, a minimum of 30 heterogeneous participants was targeted for recruitment [33,34]. This guideline is consistent with similar studies pursuing similar objectives [35].

#### 2.5.1. Data Processing and Software

All statistical analyses were conducted using IBM SPSS Statistics version 28.0. A *p*-value of *p* < 0.05 was considered statistically significant.

#### 2.5.2. Descriptive Statistics

Descriptive statistics were used to summarize participant characteristics. Given the relatively small sample size and the dominance of non-normally distributed data, non-parametric statistics were chosen. Continuous variables were reported as median with interquartile range (IQR), and categorical variables as numbers and percentages (%).

#### 2.5.3. Absolute Agreement

Bland–Altman plots with 95% limits of agreement (LOA) were created to evaluate agreement within and between raters. The Bland–Altman plots illustrate the agreement between raters and within one rater. The LOA were defined as the mean difference ±1.96 times the standard deviation of the differences. These plots provide a visual representation of agreement and potential bias between measurements.

#### 2.5.4. Reliability Analyses

To assess inter- and intra-reliability, Intraclass Correlation Coefficients (ICC^2,1^) with 95% Confidence Intervals (95% CI) were calculated using a two-way mixed-effects model for absolute agreement based on a single rating (k = 1).

#### 2.5.5. Systematic Differences

Potential systematic differences between the two testing occasions of the first rater (R1a and R1b) and between both raters (R1a and R2) were assessed using the Wilcoxon signed-rank test.

#### 2.5.6. Measurement Error

Measurement precision was quantified using the Standard Error of Measurement (SEM), calculated as follows [34]:

SEM = SD × √(1 − ICC).

where SD is the standard deviation of the differences between the two assessments. It offered insight into how much observed scores deviate from an individual’s true score due to measurement error. To allow comparison between methods, the relative SEM (%SEM) was also calculated as follows:

$$\%\mathrm{SEM}=(\frac{SEM}{mean})\;\times \;100.$$

A lower SEM or %SEM indicates higher measurement precision. The Smallest Real Difference (SRD), representing the smallest detectable change beyond measurement error, was calculated as follows [34]:

SRD = 1.96 × SEM × √2.

#### 2.5.7. Exploratory Correlation Analyses

As an exploratory analysis, Spearman correlation coefficients were calculated to examine relationships between the three assessment methods at the same measurement point (*n* = 27 comparisons). It was hypothesized that higher percentages of local tissue water would be associated with greater dermal thickness and larger circumference measurements, as all may reflect the presence of HNL. Correlation strength was interpreted as follows: below 0.40 as “weak”, values between 0.40 to 0.74 as “moderate”, values between 0.75 to 0.90 as “strong”, and values above 0.90 as “very strong” [33,34,36,37].

#### 2.5.8. Clinical Feasibility Evaluation

To assess clinical feasibility, the first rater (KVA) evaluated both “time efficiency” and “clinical limitations” of the three assessment methods. Time efficiency included the duration of preparation, execution, and data processing (measured in seconds). Medians and SDs were calculated. Clinical limitations were documented by the first rater (KVA) documented based on clinical observations. Limitations described in the literature were supplemented with insights from practical experience. An ‘X’ was marked for each observed limitation per method, and the total number of limitations was reported for each method.

## 3. Results

Thirty-three HNC patients were included, 24 of whom were male and 9 were female. The median age was 64 years. All patients’ characteristics are given in Table 2.

A cohort of 33 patients with or without HNL was included. It is important to note that not all measurement points had complete data due to certain limitations of the devices. Missing MMDC results were frequently caused by factors such as beard growth or open wounds, which can interfere with accurate readings. Similarly, technical challenges, such as difficulties in obtaining clear images at certain angles or locations, contributed to missing ultrasound data. Table 3 and Table 4 show the extent of missing data for each measurement point, while Table 5 provides a more detailed overview of the possible reasons (i.e., clinical limitations) behind the missing data for each method.

### 3.1. Reliability

**Figure 2:**
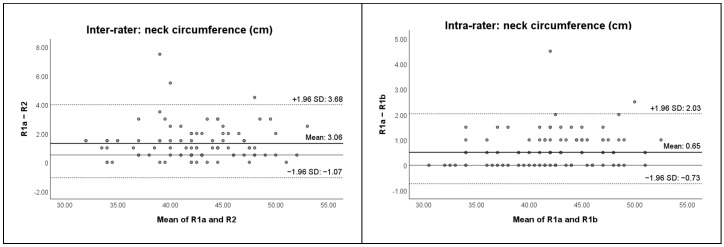

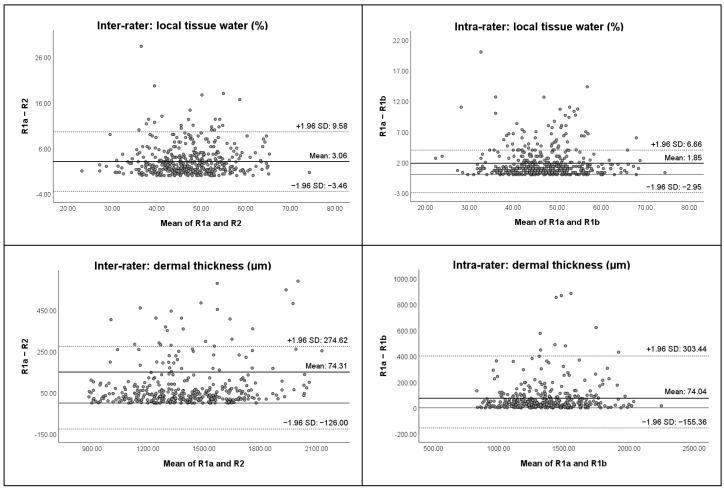
Bland–Altman plots with their 95% limits of agreement (LOA). R1a = first measurement of rater 1; R1b = second measurement of rater 1; R2 = rater 2; SD = standard deviation; the solid line represents the 0 reference line on the *y*-axis. The 95% limits of agreement are represented by horizontal dashed lines. The mean difference is shown by a horizontal bold solid line.

The Bland–Altman plots with their 95% LOA are displayed in Figure 2.

The inter- and intra-rater reliability results are shown in Table 3 and Table 4, together with the median of each measurement, the (%)SEMs and SRDs and the *p*-values of the Wilcoxon signed-rank test.

#### 3.1.1. Inter-Rater Reliability

The local tissue water assessment demonstrated moderate to very strong ICCs^2,1^ (ICCs^2,1^ from 0.644 to 0.974). The ICCs^2,1^ for the neck circumference measurements indicated very strong reliability (ICCs^2,1^ from 0.958 to 0.973). The dermal thickness measurements exhibited strong to very strong reliability (ICCs^2,1^ from 0.849 to 0.982), except one weak outlier for dermal thickness of the “Inferior Sternocleidomastoid” point left (ICC^2,1^ of 0.136). The variations in the scores between the assessments of the first and second rater are indicated by the SEMs. The SEMs for the local tissue water assessments varied from 1% to 4%, indicating a small SEM. For neck circumference measurements, all SEMs were 1.0 cm. Values ranging from 28 µm to 360 µm were observed for the measurements of dermal thickness. The %SEMs for the local tissue water assessments varied from 3% to 9%, while for the neck circumference measurements varied from 2.0% to 2.5%, and the dermal thickness measurements varied from 2% to 28%. The relatively high SRD values, along with the calculated medians for each assessment (R1a, R2), indicate that the differences between the medians cannot be attributed solely to measurement error. The median change between the assessments of both raters did not exceed the SRD, suggesting that the differences between both raters are not clinically meaningful. The Wilcoxon signed-rank test showed only a few significant differences between the raters, particularly for the local tissue water assessment and for one measurement point for dermal thickness. For inter-rater agreement, the mean differences for all three assessment methods appear small, with data points predominantly falling within the 95% LOA. However, there is some variability, particularly for local tissue water and dermal thickness, where outliers were observed.

#### 3.1.2. Intra-Rater Reliability

The local tissue water assessment demonstrated consistently very strong ICCs^2,1^ (ICCs^2,1^ from 0.851 to 0.990), except for the “Mid-Tragus-Oral” point left (ICC^2,1^ of 0.851). The ICCs^2,1^ for neck circumference measurements indicated very strong intra-rater reliability (ICCs^2,1^ from 0.982 to 0.994). The dermal thickness measurements showed strong to very strong intra-rater reliability (ICCs^2,1^ from 0.781 to 0.969), except for two outliers with weak ICCs^2,1^ of 0.354 and 0.254 for the “Temporal” point right and the “Inferior Sternocleidomastoid” point left, respectively. The variations in the scores from the first to the second assessments of the first rater are indicated by the SEMs. The SEMs for the local tissue water assessment varied from 1% to 3%, indicating a small SEM. For neck circumference measurements, all SEMs were 0.5 cm. Values ranging from 31 µm to 339 µm were observed for the measurements of dermal thickness. The %SEMs for the local tissue water assessment varied from 2% to 7%, while the neck circumference measurements varied from 1.0% to 1.5%, and the dermal thickness measurements varied from 2% to 26%. The relatively high SRD values, along with the calculated medians for each assessment (R1a, R1b), suggest that the differences between the medians might be within the range of measurement error. The median change between both assessments of the first rater did not exceed the SRD, suggesting that the differences within one rater are not clinically meaningful. The Wilcoxon signed-rank test showed only a few significant differences within one rater, particularly for local tissue water. Based on the Bland–Altman plots, for intra-rater agreement, the mean differences between the first and second assessments by the first rater are minimal for all methods. Most data points lie within the 95% LOA, indicating good consistency within one rater. However, a small number of outliers were visible, particularly for dermal thickness.

### 3.2. Clinical Feasibility

Details about time efficiency and reported clinical limitations of the different assessment methods are displayed in Table 5. Based on clinical experience and the literature, six limitations for local tissue water, three for neck circumference, and eight for dermal thickness assessments were identified.

### 3.3. Correlation Analyses

Only 2 (out of 27) correlations were significant: between local tissue water and dermal thickness of the “Mid Sternocleidomastoid” (5b) point at the right side, and between local tissue water and dermal thickness of the “Inferior Sternocleidomastoid” (5c) point at the right side as well. All found correlations were weak, except for one moderate correlation, i.e., the positive correlation between local tissue water and dermal thickness of the “Inferior Sternocleidomastoid” (5c) point at the right side. Remarkably, the correlation coefficients mainly hover around zero, indicating little to no linear relationship between the different assessment methods. The results are shown in Table 6.

## 4. Discussion

The present study showed reasonable reliability between and within raters. It also showed that local tissue water, neck circumference, and dermal thickness are poorly related concepts that probably provide complementary, rather than similar, information to clinicians evaluating patients after radiotherapy for HNC.

First, all three assessment methods demonstrated moderate to very strong reliability, except for three weak outliers for dermal thickness. In the recent study by Arends et al., a group of 50 healthy participants underwent the same protocol with multiple measurement sites as in this study, to assess the test-retest reliability of the LymphScanner, a similar device to the MMDC [21]. Our findings are consistent with those of Arends et al., who reported strong to very strong reliability, with ICC^2,1^ values ranging from 0.81 to 0.95 and minimal SEM values between 1.51 and 2.86 [21]. Both in our study and the research of Arends et al., the temporal region and the area above/below the mandible showed lower reliability, likely due to their proximity to bone structures, which may increase the impact of probe placement [21]. Previous research has also indicated that measurement points near bones and tendons tend to demonstrate lower reliability and higher measurement errors; therefore, caution should be exercised when using these sites [21,39]. It is important to note that skin conditions such as damage, eczema, active inflammation, skin folds, and scars were identified as factors that could affect the contact between the probe and the skin, potentially impacting measurement accuracy. Also, short stubbles (even after shaving) may affect the reliability of the MMDC device in men or even make measurement impossible, as illustrated by our missing data at these measurement points. This aligns with previous findings suggesting that variation in measurements could be influenced by sex [21,40,41]. In addition to the influence of facial hair, men also tend to have thicker facial skin [42,43], contributing to variability in measurements and potentially affecting the precision of the MMDC device in male patients. Our results show stronger intra-rater reliability than inter-rater reliability. One possible explanation is the potential inconsistency in MMDC device placement between different raters. As noted in previous research, slight variations in device positioning can lead to significant differences [21]. Similar to the studies conducted by Chotipanich et al. and Purcell et al., a high level of reliability was observed in neck circumference measurements (ICCs^2,1^ higher than 0.90) [18,22,38]. This could be attributed to the simplicity of performing neck circumference measurements, which require minimal anatomical expertise to identify landmarks accurately. Additionally, due to the relatively stable shape of the neck, variations in tape placement from the optimal position have minimal impact on circumference measurements. However, it is worth noting that movements of the head and neck by subjects during measurements may lead to slight shifts in the location of landmarks. Regarding the reliability of dermal thickness measurement, no other studies in the head and neck region were found. In the breast cancer population, dermal thickness as a measure for breast edema has been studied before [24,26,27]. Our reliability findings are consistent with previous research in breast cancer patients [24,26,27]. Our results showed strong to very strong reliability, with the “Temporal” and “Inferior Sternocleidomastoid” points as exceptions, showing weak reliability. The naturally thin skin in the “Temporal” area might make it difficult for ultrasound to clearly differentiate the dermal layer from the epidermis and subcutis, potentially leading to inaccurate measurements. The complex anatomical location of the “Inferior Sternocleidomastoid” and the variation in neck position and patient anatomy further complicate the visualization of the underlying tissues. In addition, as mentioned above, men tend to have thicker facial skin compared to women [42,43]. Therefore, the ultrasound measurement of facial skin thickness should be interpreted in relation to sex. For all three assessment methods, the %SEMs were consistently higher between raters compared to within a single rater, indicating greater variability in measurements between different evaluators. The %SEM findings indicate that the variability in measurements differs across the three methods, with neck circumference measurements showing the least variability both within and between raters. Dermal thickness measurements, however, exhibited the highest variability, particularly between raters. The Bland–Altman plots indicate overall acceptable agreement for both inter- and intra-rater assessments, with some variability in local tissue water and dermal thickness measurements.

Second, clinical feasibility was evaluated by considering time efficiency and reported clinical limitations. As far as we know, the clinical feasibility of these methods has not yet been investigated before in this population. Based on our study, each assessment method appears to have its own specific advantages and disadvantages. The MMDC proved to be a highly reliable device. However, it often provided inaccurate readings when participants had wounds, dehydrated skin, possible fibrosis, scars, or stubbles, possibly making some measurements less reliable. The missing data range of 3% to 18% further supports this inconsistency, suggesting that its daily clinical application might be limited, as these conditions are common in clinical practice. The neck circumference measurement was the fastest method, followed by the MMDC. In this, it is important to note that the neck circumference measurement involves only 3 measurement points at the level of the neck compared to 15 in the head and neck area for the other methods. Also, the tension of the tape measure on the skin is not standardized. In addition, it does not provide information on underlying tissue changes, such as fluid composition. So, it cannot differentiate between fat accumulation, muscle hypertrophy, and true lymphedema. It is crucial to also highlight that the three measurement points provide only an indication of lymphedema in the neck region, not in the face. Regarding the dermal thickness assessments with ultrasound, most limitations were noted. Opting for the small probe was deemed preferable for facial measurements. Although the large probe offers superior and more detailed images, practical considerations, such as participant discomfort and excessive facial space, led to the selection of the small probe. Dermal thickness measurements were particularly sensitive to missing data, which further limits their feasibility for use in clinical practice. When processing the ultrasound images with ‘Weasis’, it is important to note some limitations: it is a very time-consuming process and the program’s efficiency is not yet optimal. Ultrasound assessments require specific expertise from clinicians as well.

Regarding the correlation analyses, weak to moderate correlations were found between all three assessment methods. To explain the low correlations between the three HNL assessment methods, it is important to consider how each of these methods measures different physiological aspects related to HNL. Also, the measured location and measurement depth are different for these three methods. While the MMDC primarily assesses the dermis and part of the (upper) subcutis, depending on individual skin thickness, the dermal thickness method assesses only the dermis. The MMDC does not assess deeper tissues, limiting its ability to evaluate the full extent of HNL. Unlike in the arms or legs, where circumferential or volume measurements could be considered a gold standard for assessing swelling [23], applying such methods in the head and neck region is far more challenging. As a result, the methods investigated in this study are better suited for assessing and monitoring “swelling” rather than definitively diagnosing HNL. While we did indeed not expect high correlations due to the different constructs being assessed, some degree of relationship among the methods was anticipated, especially between the MMDC and dermal thickness. There are different reasons for these unexpected findings. First, the MMDC assesses the moisture content of the skin, which can indirectly indicate fluid retention. However, skin moisture levels can also be affected by factors like skin hydration status, local inflammation, or skin conditions that are unrelated to lymphedema [44]. Second, the ultrasound measurement provides a direct measurement of dermal thickness. While increased dermal thickness can be a sign of lymphedema, it can also be influenced by fibrosis, inflammation, or other changes in tissue composition that are not necessarily related to fluid buildup. Third, the neck circumference measurement evaluates the overall swelling or volume increase in the neck area. However, it cannot distinguish between lymphedema caused by fluid buildup and other factors contributing to increased circumference, such as muscle hypertrophy, fibrosis, scars, subcutaneous fat increase in obese individuals, or biological gender differences, with men typically having higher values [45]. In conclusion, based on this exploratory correlation analysis, each of these assessment methods seems to evaluate different physiological features of HNL.

Although the study sample included patients treated with either primary (C)RT or post-operative (C)RT, and with varying time intervals since treatment, this heterogeneity is acknowledged. Since lymphedema can develop in both patient groups, and the primary aim was to assess the reliability and clinical feasibility of different assessment methods (rather than to compare outcomes across subgroups), no subgroup distinction was made. Moreover, since reliability analyses were based on repeated measurements within the same individuals, differences in treatment modality or time since treatment do not influence the reliability outcomes. Furthermore, including a heterogeneous sample enhances the generalizability of the findings to the broader HNC population at risk for developing HNL. While this study focused on the reliability and feasibility of assessment methods, future research should consider incorporating treatment-related variables, such as the radiation field and dosage, to improve clinical applicability. This research exhibits several strengths. First, a notable strength of this study is that the measurements were conducted in real-world conditions, rather than in an artificial laboratory setting. This approach better reflects the challenges and pressures encountered in everyday clinical practice. Second, a standardized protocol was used to ensure accurate and reliable data across different raters and over time [21]. This specification regarding patient positioning and the predetermined measurement points likely contributed to the strong reliability. Third, the combined investigation of intra-rater and inter-rater reliability, along with clinical feasibility, is another advantage. A comprehensive evaluation of the reliability of the three assessment methods was conducted using the ICC^2,1^ with 95% CI, as well as the SEM, the %SEM, the SRD, the Wilcoxon signed-rank test, and the Bland–Altman plots with their 95% LOA. Fourth, measurements conducted by both raters were scheduled within the same consultation to account for the day-to-day variability of lymphedema. Previous research in patients with breast cancer-related lymphedema has shown that this variability can have various causes, such as fluid retention, temperature, activity level, and other physiological factors [46,47,48]. Therefore, we aimed to obtain more consistent data by minimizing the influence of daily fluctuations. Furthermore, this approach provided the opportunity to make direct comparisons between the measurements, resulting in a more accurate understanding of any correlations between the different assessments. Similar to the previous research by Arends et al., we also conducted triplicate measurements instead of taking single measurements for local tissue water. Given the minimal additional time required, this approach appears justified due to its significant improvement in reliability [21]. Next, our results were derived from measurements on an HNC population, both with and without HNL. This could be considered a limitation, as our sample includes participants who may not have lymphedema, which could potentially reduce the variability in our outcome measures, making it more challenging to achieve high reliability. However, it is also a strength, as it extends research into the HNC population, rather than being limited to studies involving only healthy participants. This broader scope allows for a more comprehensive understanding of the measurement’s applicability and reliability in a clinical population at risk for developing lymphedema.

Our study also has a few limitations. First, regarding the dermal thickness assessment, because of feasibility reasons, all data processing was conducted afterward by the same first rater, regardless of who acquired the ultrasound images. Second, at various measurement points, our study included differing numbers of participants, which may result in a biased picture. This variance in participant numbers is primarily attributed to missing data, which can arise from beard growth, stubbles, open wounds or dry skin (as side effects from the cancer treatment). Finally, correlations between the different assessment methods were only evaluated through correlation analyses using Spearman correlation coefficients. Since no gold standard currently exists for identifying the presence of HNL, we did not investigate concurrent or known-groups validity. Advanced imaging techniques, such as Computed Tomography (CT) and Magnetic Resonance Imaging (MRI), may serve as potential reference standards in future validation studies. While their routine clinical use remains limited by factors such as cost, time, and, in the case of CT, radiation exposure, they remain promising tools for research [14,19]. The CT Lymphedema and Fibrosis Assessment Tool (CT-LEFAT), developed to standardize the diagnosis of lymphedema and fibrosis based on fat stranding as a marker of HNL on CT, demonstrated low inter- and intra-rater reliability [49,50]. This highlights human performance as a limiting factor and suggests that further refinement, training, and potential integration of artificial intelligence are necessary to enhance its diagnostic accuracy [50]. Despite minimal application in HNL to date, MRI has demonstrated strong utility in extremity lymphedema, particularly in differentiating between fluid and fibrotic tissue, which underscores its potential value for head and neck applications as well [14,51,52]. Further validation of both modalities is essential to support their use as objective diagnostic tools.

## 5. Conclusions

This study highlights the need for continued research to enhance the detection, monitoring, and management of HNL in the HNC population, aiming to develop more refined assessment tools that can accurately capture small fluctuations over time. Therefore, a comprehensive validation study in a large sample with HNC patients with and without HNL, potentially using advanced techniques like MRI or CT as a reference, is needed to establish a standard for assessing HNL. Further investigation is needed to assess the value of facial distances/circumferences for detecting and evaluating HNL. These measurements could possibly offer a practical, cost-effective tool in clinical settings, as they do not require additional equipment and can be easily implemented in routine practice. Future research should focus on establishing normal cut-off values for these outcomes as well, as this could provide a crucial benchmark for more accurate assessment and diagnosis of the condition. In this context, it is important to take into account that different skin types (e.g., dry, oily, and combination), ethnicities, body mass indices, and genders can have different baseline levels of local tissue water, which could make comparisons between individuals difficult. Future research should primarily focus on concurrent validity by comparing these assessment methods to a gold standard, in order to investigate their accuracy in assessing HNL. This approach will help confirm whether these methods provide reliable evaluations of lymphedema. Future research should employ longitudinal designs with baseline assessment prior to cancer treatment to evaluate the ability of these three assessment methods to detect changes in lymphedema over time, addressing the limitation of the cross-sectional approach in our study. However, patient-reported assessments should also be integrated with objective methods to evaluate symptom burden and track improvements over time.

Given the complexity of HNL and its significant impact, a multifactorial assessment approach seems warranted. However, we acknowledge that the combined daily use of all three assessment methods may not be practical in routine clinical settings. A tailored selection of tools based on clinical priorities and feasibility is therefore advised. Importantly, the aim of this study was not to evaluate the combined reliability of these methods, but to assess each tool individually. Given the differences in the constructs being measured, future validity studies will be needed to determine which combination of assessment methods is most meaningful and feasible for daily clinical use. In conclusion, when considering our reliability and clinical feasibility findings, the neck circumference measurement proved to be the most reliable and time-efficient method for assessing lymphedema in the neck area. For the head, local tissue water assessment seems the most reliable and feasible. For evaluation and follow-up of the neck volume, tape measurements of neck circumference are recommended at the superior, mid, and inferior sternocleidomastoid measurement points. The measurement error is very small and varies between 1.0% and 2.5%. For the head, assessment of local tissue water seems to be the most reliable and feasible. For evaluation and follow-up of lymphedema of the head and neck, MMDC measurements determining the percentage of local tissue water at the temporal, mid-tragus-oral, superior and inferior mandibular, submental, superior, mid, and inferior sternocleidomastoid measurement points are recommended. Measurement error is small and varies between 2% and 9%. For evaluation and follow-up of lymphedema of the head and neck, the dermal thickness can be determined by using ultrasound at the mid-tragus-oral, superior and inferior mandibular, submental, and superior and mid sternocleidomastoid measurement points. Measurement error ranges from 2% to 28%, indicating some variability in the accuracy of measurements at the temporal and inferior sternocleidomastoid measurement points. Further research should confirm how these methods can complement each another.

## Figures and Tables

**Table 1:** Overview of the measurement points in the head and neck region per assessment method [21].

Assessment Methods	Measurement Points	
% local tissue water with the MMDC Dermal thickness with B-mode ultrasound	1—“Temporal” point left and right 2—“Mid-Tragus-Oral” point left and right 3a—“Superior Mandibular” point left and right 3b—“Inferior Mandibular” point left and right 4—“Submental” point 5a—“Superior Sternocleidomastoid” point left and right 5b—“Mid Sternocleidomastoid” point left and right 5c—“Inferior Sternocleidomastoid” point left and right	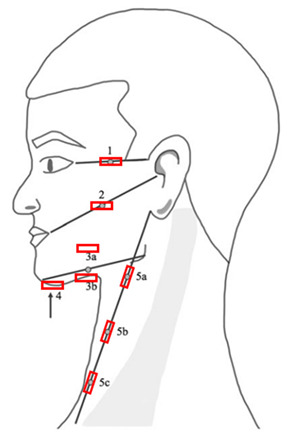
Neck circumference with a tape measure	5a—“Superior Sternocleidomastoid” point 5b—“Mid Sternocleidomastoid” point 5c—“Inferior Sternocleidomastoid” point	

MMDC = MoistureMeterD Compact; anatomical reference names were assigned to each measurement point, based on their positions relative to specific anatomical landmarks. 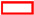 = positioning of the probes, for the measurement of dermal thickness with B-mode ultrasound. Measurement points were marked with a washable skin pencil as follows: from the lateral corner of the eye to the top of the ear (point 1); from the corner of the mouth to the tragus (point 2); and from the chin to the jaw, with the MMDC placed 1 cm above (point 3a); and 1 cm below (point 3b). The submental area was marked centrally (point 4). The tape measure was placed from the earlobe to the sternoclavicular joint, with points 5a, 5b, and 5c marked at 25%, 50%, and 75% of the total distance, respectively.

**Table 2:** Descriptive statistics of the characteristics of the sample (*n* = 33).

	Median (IQR) [Min–Max]	
Age (years)	64.0 (52.5–69.5) [29–84]	
BMI (kg/m^2^)	26.9 (23.6–30.5) [12.7–37.3]	
Time since initiation of radiotherapy (weeks)	24 (12–52) [6–52]	
		Number (%)
Gender	Male	24 (73%)
	Female	9 (27%)
Skin type	White	32 (97%)
	Other	1 (3%)
Primary tumor location	Nasal cavity/paranasal sinuses	5 (15%)
	Oropharynx	6 (18%)
	Oral cavity	11 (33%)
	Hypopharynx	2 (6%)
	Larynx	2 (6%)
	Salivary gland	4 (12%)
	Thyroid	1 (3%)
	Other	2 (6%)
T-Tumor classification (TNM) [32]	T1	7 (21%)
	T2	9 (27%)
	T3	6 (18%)
	T4	10 (30%)
	TX	1 (3%)
N-Node classification (TNM) [32]	N0	16 (48%)
	N1	7 (21%)
	N2	8 (27%)
	NX	2 (6%)
Cancer treatment	Definitive (C)RT	19 (58%)
	Unilateral RT	7 (21%)
	Bilateral RT	3 (9%)
	Unilateral CRT	6 (18%)
	Bilateral CRT	3 (9%)
	Post-operative (C)RT	14 (42%)
	Unilateral neck dissection	9 (27%)
	Bilateral neck dissection	5 (15%)
Presence of HNL based on a positive response to item 6 of the Lymphedema Symptom Intensity and Distress Survey-Head and Neck version 2.0: “Swelling in your face, head or neck?”	Subjective presence of HNL	15 (45%)
Presence of HNL based on visual inspection	Subjective presence of HNL	19 (58%)

IQR = interquartile range; BMI = body mass index; (C)RT = (chemo)radiotherapy; HNL = head and neck lymphedema.

**Table 3:** Median and Wilcoxon signed-rank test of the three assessment methods.

		Median (Q1, Q3)						*p*-Value Wilcoxon Signed-Rank Test	
		R1a	N	R1b	N	R2	N	Inter-Rater	Intra-Rater
Local tissue water (in %)									
1 “Temporal”	R	43 (40, 48)	32	44 (41, 50)	31	45 (41, 49)	33	0.125	0.167
	L	43 (39, 46)	32	44 (38, 48)	31	45 (40, 47)	33	0.085	0.095
2 “Mid-Tragus-Oral”	R	46 (40, 50)	30	46 (41, 51)	29	46 (41, 51)	32	0.914	0.248
	L	42 (38, 46)	30	42 (40, 47)	29	44 (39, 48)	31	0.416	**0.046**
3a “Superior Mandibular”	R	44 (40, 48)	30	45 (39, 48)	29	47 (41, 52)	27	0.265	0.152
	L	45 (40, 47)	28	45 (41, 49)	27	44 (40, 49)	31	0.973	**0.037**
3b “Inferior Mandibular”	R	48 (44, 52)	30	48 (41, 51)	30	48 (43, 52)	31	0.428	0.260
	L	46 (44, 51)	29	47 (44, 54)	27	45 (42, 52)	30	0.107	0.466
4 “Submental”	n/a	44 (41, 50)	31	44 (40, 49)	29	45 (40, 50)	32	0.934	0.798
5a “Superior SCM”	R	48 (44, 53)	31	50 (45, 55)	30	48 (43, 53)	32	0.829	**0.028**
	L	43 (38, 46)	31	48 (43, 53)	30	50 (46, 55)	32	**0.034**	**0.030**
5b “Mid SCM”	R	50 (68, 56)	31	52 (48, 56)	30	52 (48, 57)	32	0.617	0.284
	L	50 (46, 56)	31	51 (46, 58)	30	52 (48, 55)	31	0.496	**0.007**
5c “Inferior SCM”	R	49 (42, 54)	32	49 (43, 53)	30	52 (48, 56)	33	0.277	0.278
	L	51 (46, 53)	31	50 (46, 53)	31	48 (44, 52)	33	**0.008**	0.329
Neck circumference (in cm)									
5a “Superior SCM”	n/a	43.5 (42.0, 47.5)	31	42.5 (41.5, 47.5)	31	44.0 (40.0, 47.5)	33	0.885	0.102
5b “Mid SCM”	n/a	42.0 (39.5, 44.0)	31	42.0 (39.0, 45.0)	31	42.5 (39.0, 45.0)	33	0.195	0.372
5c “Inferior SCM”	n/a	42.5 (39.0, 45.5)	31	42.5 (39.0, 45.0)	31	42.5 (39.5, 46.0)	33	0.609	0.562
Dermal thickness (in µm)									
1 “Temporal”	R	1403 (1209, 1654)	28	1418 (1220, 1784)	25	1480 (1245, 1721)	26	0.288	0.118
	L	1415 (1109, 1647)	28	1338 (1113, 1628)	25	1413 (1068, 1721)	26	0.459	0.581
2 “Mid-Tragus-Oral”	R	1342 (1159, 1670)	28	1412 (1174, 1693)	25	1419 (1186, 1685)	26	0.534	0.701
	L	1418 (1067, 1657)	28	1418 (1121, 1567)	25	1459 (1094, 1625)	26	0.280	0.532
3a “Superior Mandibular”	R	1250 (1189, 1474)	28	1256 (1174, 1487)	25	1288 (1203, 1432)	26	0.638	0.809
	L	1296 (1153, 1412)	28	1279 (1141, 1358)	25	1276 (1156, 1358)	26	**0.048**	0.269
3b “Inferior Mandibular”	R	1456 (1281, 1631)	28	1494 (1252, 1630)	25	1469 (1345, 1593)	26	0.265	0.889
	L	1417 (1253, 1641)	28	1379 (1231, 1612)	25	1469 (1237, 1621)	26	0.694	0.597
4 “Submental”	n/a	1357 (1220, 1494)	28	1433 (1189, 1528)	25	1404 (1250, 1566)	26	0.638	0.895
5a “Superior SCM”	R	1436 (1241, 1569)	28	1482 (1243, 1582)	25	1428 (1227, 1578)	26	0.968	0.882
	L	1408 (1243, 1552)	28	1389 (1236, 1515)	25	1503 (1250, 1584)	26	0.932	0.220
5b “Mid SCM”	R	1278 (1032, 1525)	28	1316 (1125, 1539)	25	1358 (1194, 1491)	26	0.737	0.220
	L	1301 (1202, 1483)	28	1284 (1174, 1416)	25	1351 (1139, 1570)	26	0.182	0.115
5c “Inferior SCM”	R	1242 (1016, 1401)	28	1247 (1031, 1391)	25	1282 (1086, 1385)	26	0.770	0.829
	L	1281 (1067, 1397)	28	1249 (1117, 1377)	25	1286 (1104, 1425)	26	0.454	0.166

Q1 = first quartile; Q3 = third quartile; R1a = first measurement of rater 1; R1b = second measurement of rater 1; R2 = rater 2; R = right side; L = left side; SCM = sternocleidomastoid; ***p*-value < 0.05: significant (Wilcoxon)**.

**Table 4:** Inter- and intra-rater reliability of the three assessment methods.

		Inter-Rater				Intra-Rater			
		ICC^2,1^ (95% CI)	SEM	%SEM	SRD	ICC^2,1^ (95% CI)	SEM	%SEM	SRD
Local tissue water (in %)									
1 “Temporal”	R	0.727 (0.448–0.865)	3	7	8	0.919 (0.836–0.960)	2	4	5
	L	0.813 (0.625–0.907)	2	6	8	0.963 (0.923–0.982)	1	3	4
2 “Mid-Tragus-Oral”	R	0.882 (0.754–0.944)	2	5	6	0.922 (0.839–0.962)	2	4	5
	L	0.774 (0.514–0.894)	4	9	11	0.851 (0.691–0.929)	3	7	8
3a “Superior Mandibular”	R	0.644 (0.254–0.833)	4	9	12	0.914 (0.819–0.960)	2	5	6
	L	0.745 (0.454–0.880)	3	8	9	0.908 (0.798–0.957)	2	4	5
3b “Inferior Mandibular”	R	0.951 (0.900–0.976)	2	4	6	0.990 (0.979–0.995)	1	2	3
	L	0.938 (0.872–0.971)	2	4	6	0.970 (0.937–0.986)	2	3	4
4 “Submental”	n/a	0.964 (0.924–0.982)	1	3	4	0.939 (0.875–0.971)	2	4	6
5a “Superior SCM”	R	0.906 (0.808–0.954)	2	4	6	0.974 (0.947–0.987)	1	2	3
	L	0.939 (0.874–0.970)	2	4	5	0.970 (0.939–0.986)	1	3	4
5b “Mid SCM”	R	0.938 (0.873–0.970)	2	4	5	0.988 (0.974–0.995)	1	2	2
	L	0.949 (0.885–0.976)	2	3	4	0.961 (0.908–0.982)	1	3	4
5c “Inferior SCM”	R	0.924 (0.845–0.962)	2	4	6	0.974 (0.948–0.987)	1	3	4
	L	0.888 (0.768–0.945)	2	4	5	0.972 (0.943–0.986)	1	2	3
Neck circumference (in cm)									
5a “Superior SCM”	n/a	0.958 (0.915–0.980)	1.0	2.5	3.0	0.982 (0.963–0.992)	0.5	1.5	2.0
5b “Mid SCM”	n/a	0.973 (0.945–0.987)	1.0	2.0	2.0	0.993 (0.986–0.997)	0.5	1.0	1.0
5c “Inferior SCM”	n/a	0.959 (0.916–0.980)	1.0	2.5	2.5	0.994 (0.988–0.997)	0.5	1.0	1.0
Dermal thickness (in µm)									
1 “Temporal”	R	0.956 (0.902–0.981)	67	5	186	0.354 (−0.392–0.703)	253	16	703
	L	0.945 (0.877–0.975)	82	6	227	0.969 (0.932–0.986)	58	4	161
2 “Mid-Tragus-Oral”	R	0.964 (0.921–0.984)	60	4	167	0.781 (0.518–0.900)	159	11	442
	L	0.958 (0.907–0.981)	70	5	195	0.968 (0.931–0.986)	57	4	160
3a “Superior Mandibular”	R	0.849 (0.662–0.933)	69	5	191	0.852 (0.680–0.932)	71	5	198
	L	0.860 (0.690–0.937)	63	5	174	0.969 (0.932–0.986)	31	2	86
3b “Inferior Mandibular”	R	0.900 (0.772–0.956)	69	5	191	0.915 (0.814–0.961)	70	5	194
	L	0.982 (0.960–0.992)	28	2	79	0.941 (0.870–0.973)	56	4	156
4 “Submental”	n/a	0.950 (0.890–0.977)	48	3	133	0.879 (0.735–0.945)	73	5	202
5a “Superior SCM”	R	0.903 (0.782–0.956)	76	5	124	0.840 (0.652–0.927)	100	7	277
	L	0.968 (0.928–0.986)	45	3	186	0.964 (0.922–0.984)	45	3	124
5b “Mid SCM”	R	0.921 (0.825–0.965)	70	5	194	0.896 (0.775–0.953)	77	6	212
	L	0.916 (0.813–0.962)	67	5	997	0.913 (0.802–0.961)	65	5	180
5c “Inferior SCM”	R	0.946 (0.878–0.976)	55	4	152	0.948 (0.887–0.977)	56	5	154
	L	0.136 (−0.906–0.611)	360	28	1906	0.254 (−0.617–0.658)	339	26	939

ICC = intraclass correlation coefficient; CI = confidence interval; SEM = standard error of measurement; %SEM = relative SEM; SRD = smallest real difference; R = right side; L = left side; SCM = sternocleidomastoid; ICC ≥ 0.90: very strong reliability; ICC ≥ 0.75: strong reliability; 0.50 ≤ ICC < 0.75: moderate reliability; ICC < 0.50: weak reliability.

**Table 5:** Time efficiency and clinical limitations of the different assessment methods (for all measurement points).

	Local Tissue Water	Neck Circumference	Dermal Thickness
Time efficiency (in seconds)			
Median preparation time (SD)	153 (15)	21 (6)	153 (15)
Median execution time (SD)	405 (70)	39 (8)	557 (81)
Median process time (SD)	n/a	n/a	1778 (124)
Median total time (SD)	579 (71)	57 (11)	2476 (257)
Clinical limitations			
Training of raters is needed. “The quality and accuracy of the measurements can vary significantly based on the raters’ skill and experience.” “The head and neck area is anatomically complex with many curves and contours. Achieving consistent contact with the device on curved surfaces like the jawline or chin can be challenging, potentially leading to variations in readings.” [21] “A discrepancy between evaluators could arise if the tape measure is pulled at different degrees of tension.” [38] “Variability in how the MMDC and ultrasound probes are placed, the angle of contact and the pressure applied to the skin during the measurement can affect the results.” [21]	X	X	X
No direct assessment of HNL, measures only an aspect of potential HNL. “In areas where the skin is thicker or where subcutaneous tissues are deeper, such as certain parts of the neck, the device may not provide an accurate reflection of lymphedema throughout the entire subcutaneous layer.” “It does not provide information on underlying tissue changes, such as fluid composition, and cannot differentiate between fat accumulation, muscle hypertrophy, and true lymphedema.”	X	X	X
Effect of movement in the head and neck area. “Any movement can affect the precision and repeatability of the measurements. Repeated measurements can show variability due to slight changes in device positioning or the patients’ movements (for example breathing, swallowing). This can make it difficult to obtain highly precise or reproducible measurements, especially in the head and neck area where movement is common.”	X	X	X
“Very sensitive device. The measurement is highly sensitive to surface conditions, such as the presence of sweat on the skin, open wounds and beard growth. This might lead to missing data.”	X		X
“Changes in room temperature or skin temperature during measurement can introduce variability, especially since the skin on the face and neck is sensitive to temperature changes.” [21]	X		X
“The device can be sensitive to the presence of veins and other blood vessels beneath the skin. The results are also affected by other factors like blood flow and vessel density.”	X		
“Expensive device because high-frequency ultrasound is needed to achieve detailed images of thin structures like the dermis.”			X
“The device is not portable or easily moved.”			X
“In cases of severe HNL, it can be difficult to distinguish between the dermis and subcutis on ultrasound.”			X
Number of limitations	6	3	8

SD = standard deviation; MMDC = MoistureMeterD Compact; HNL = head and neck lymphedema.

**Table 6:** Correlations between the three assessment methods using Spearman correlation coefficients.

Expected Correlations	R	L
1. Positive correlation between local tissue water and dermal thickness of the “Temporal” (1) point	0.076 (N = 29)	0.310 (N = 29)
2. Positive correlation between local tissue water and dermal thickness of the “Mid-Tragus-Oral” (2) point	0.143 (N = 28)	0.246 (N = 27)
3. Positive correlation between local tissue water and dermal thickness of the “Superior Mandibular” (3a) point	0.095 (N = 26)	0.267 (N = 25)
4. Positive correlation between local tissue water and dermal thickness of the “Inferior Mandibular” (3b) point	0.143 (N = 27)	0.377 (N = 27)
5. Positive correlation between local tissue water and dermal thickness of the “Submental” (4) point	0.232 (N = 28)	
6. Positive correlation between local tissue water and neck circumference of the “Superior Sternocleidomastoid” (5a) point	0.067 (N = 33)	0.229 (N = 33)
7. Positive correlation between local tissue water and dermal thickness of the “Superior Sternocleidomastoid” (5a) point	0.266 (N = 28)	0.133 (N = 28)
8. Positive correlation between the neck circumference and dermal thickness of the “Superior Sternocleidomastoid” (5a) point	0.240 (N = 29)	−0.008 (N = 29)
9.Positive correlation between local tissue water and neck circumference of the “Mid Sternocleidomastoid” (5b) point	0.192 (N = 33)	0.155 (N = 33)
10.Positive correlation between local tissue water and dermal thickness of the “Mid Sternocleidomastoid” (5b) point	0.390 * (N = 28)	0.352 (N = 28)
11. Positive correlation between the neck circumference and dermal thickness of the “Mid Sternocleidomastoid” (5b) point	0.165 (N = 29)	0.208 (N 29)
12. Positive correlation between local tissue water and neck circumference of the “Inferior Sternocleidomastoid” (5c) point	0.092 (N = 34)	0.140 (N = 34)
13. Positive correlation between local tissue water and dermal thickness of the “Inferior Sternocleidomastoid” (5c) point	0.500 **(N = 29)	0.268 (N = 29)
14. Positive correlation between the neck circumference and dermal thickness of the “Inferior Sternocleidomastoid” (5c) point	0.033 (N = 29)	−0.270 (N = 29)

R = right side; L = left side; N = number; MMDC = MoistureMeterD Compact; 0.50 ≤ correlation < 0.75: moderate; correlation < 0.50: weak; ** = correlation is significant at the 0.01 level (2-tailed); * = correlation is significant at the 0.05 level (2-tailed); with hypothesis **accepted** and hypothesis rejected at the 0.5 threshold.

## Data Availability

The datasets presented in this article are not readily available because the data are part of an ongoing study. Requests to access the datasets should be directed to the corresponding author.
